# Spontaneous spinal hematomas: A case series

**DOI:** 10.1007/s00701-024-06240-6

**Published:** 2024-08-28

**Authors:** Carolin Albrecht, Tobias Boeckh-Behrens, Julian Schwarting, Maria Wostrack, Bernhard Meyer, Ann-Kathrin Joerger

**Affiliations:** 1 Department of Neurosurgery, Klinikum Rechts der Isar, Technical University Munich, Munich, Germany; 2 Department of Neuroradiology, Klinikum Rechts der Isar, Technical University Munich, Munich, Germany

**Keywords:** Spontaneous spinal hematoma; subarachnoid hemorrhage; spinal arteriovenous malformation; digital subtraction angiography

## Abstract

**Purpose:**

Spontaneous spinal hematoma (SSH), a rare neurological disorder, demands immediate diagnostic evaluation and intervention to prevent lasting deficits. This case series analyzes instances, particularly highlighting cases where vascular causes were identified despite inconclusive initial imaging.

**Methods:**

In a retrospective study of 20 patients treated for SSH at a Level I spine center from 01/01/2017 to 11/15/2023, we examined demographics, clinical presentation, imaging, and treatment details. Excluding traumatic cases, we present 4 instances of SSH associated with diverse vascular pathologies.

**Results:**

Patient ages ranged from 39 to 85 years, with a median age of 66 years. 45% were male, and 55% were female. Among 20 cases, 14 were epidural hematomas, 4 subdural, 1 combined epidural and subdural, and 1 subarachnoid hemorrhage. 85% presented with neurological deficits, while 3 solely had pain-related symptoms. 55% were under anticoagulant medication, and vascular anomalies were found in 25% of cases. The cause of SSH remained unclear in 20% of cases. MRI was performed for all patients, and DSA was conducted in 25% of cases. The 4 highlighted cases involved individuals with distinct vascular pathologies managed surgically.

**Conclusion:**

Urgent attention is crucial for SSH due to possible lasting neurological consequences. The study emphasizes comprehensive diagnostics and surgical exploration, especially in cases with unclear etiology, to identify and address vascular causes, preventing hematoma progression or recurrence. Despite their rarity, vascular malformations contributing to spinal hematomas warrant particular attention.

## Background

Spontaneous spinal hematoma is a very rare yet serious neurological disorder that necessitates immediate diagnostic assessment and treatment [12]. In the absence of timely and appropriate intervention, it frequently results in enduring neurological impairment [12]. Spinal epidural, subdural and subarachnoid hematomas have been described [12]. The reported incidence of spontaneous spinal epidural hematomas is 0.1 cases per 100,000 individuals annually [9]. Groen and Ponssen conducted one of the largest reviews of spontaneous spinal epidural hematomas, providing significant insights into the epidemiology and clinical presentation. Analyzing 199 cases reported between 1869 and 1989, they found that most hemorrhages occurred in the cervicothoracic region, with a mean patient age of 42 years and a slight male predominance [8]. Spontaneous spinal subdural and subarachnoid hematomas are even rarer. In a recent review of literature, only 122 published cases of spontaneous spinal subdural hematoma were described in the period from 1948 to 2014 [4]. A review of 106 cases of spinal subdural hematomas by Domenicucci et al*.* offers a thorough overview of this condition's etiologies and clinical presentations. They identified iatrogenic causes, such as lumbar puncture, as the most common, followed by coagulopathy [5].

For spontaneous spinal subarachnoid hematoma only case reports and case series of small numbers exist. However, in recent times, the increased utilization of advanced imaging techniques has likely led to a higher rate of detection of spinal hematomas [10].

Spinal epidural and subdural hematomas often manifest with intense, lancinating pain at the site of the hemorrhage. This pain may be followed, in some instances, by a pain-free interval lasting from minutes to days [12]. Subsequently, progressive neurological deficits emerge, affecting motor function, sensory perception, as well as bladder and bowel control below the spinal level affected [26]. In contrast, spinal subarachnoid hematoma can cause symptoms resembling meningitis, altered consciousness, and epileptic seizures [12].

Various factors may contribute to the development of spontaneous spinal hematomas, including anticoagulant therapy, coagulation disorders, labile hypertension, structural vascular anomalies and vasculitis, tumors, pregnancy and spinal epidural anesthesia procedures in combination with anticoagulant therapy [12, 21, 24–26]. In as many as 30 – 40% of instances of spinal hematoma, the origin of the bleeding cannot be definitively determined [4, 12].

Therapeutic options for spinal hemorrhage include conservative and surgical treatments, depending primarily on the cause and the patient's neurological status.

In 2004, Liao et al*.* [16] retrospectively reviewed 35 patients with spontaneous spinal epidural hematoma to evaluate treatment outcomes and predisposing factors. They analyzed age, sex, hypertension, anticoagulation history, and neurological outcomes using a standardized grading system. Surgery was found to be safe and effective, with a 5.7% disease-related mortality rate and a 2.9% complication rate. Patients with incomplete preoperative deficits had significantly better recovery (88.9%) than those with complete deficits (37.5%). Early surgery (within 48 h) and shorter duration of complete neurological symptoms (< 12 h) were associated with better recovery.

Another study by Liao et al*.* [15], conducted in 2009, evaluated outcomes for seventeen patients with spontaneous spinal epidural hematomas. The study again found that patients with incomplete deficits had significantly better outcomes and faster recovery (achieving functional independence within a month) than those with complete deficits. Factors such as coagulopathy, larger hematoma size, and complete preoperative spinal dysfunction were linked to poorer postoperative recovery. The study concluded that prompt surgical evacuation, particularly in incomplete spinal cord dysfunction cases, improves outcomes, especially in the cervical and thoracic regions.

In this case series, we present 20 cases of spontaneous spinal hemorrhages in our department. The special focus was on cases where, despite initially not being clearly identified by preoperative imaging, vascular structures were found to be the cause of the bleeding, which we present in the form of exemplary case reports.

## Methods

### Study design

This retrospective cohort study was designed in accordance with the STROBE guidelines[22]. We retrospectively examined the medical data of all patients who were treated at our center upon spinal hemorrhage between January 2017 and November 2023.

Patients were selected for analysis based on the following criteria:

- Admission with spontaneous spinal hematoma from 01/01/2017 – 11/15/2023
- No trauma history as a possible cause of spinal hematoma
- Complete data set
- Written patient consent for the respective case report

### Patient data

We conducted a retrospective study comprising of consecutive patients treated for spinal hematoma at a high-volume Level I surgical spine center between 01/01/2017 and 11/15/2023. Cases were retrieved from the hospital’s database by screening for the International Classification of Diseases, Tenth Revision (ICD-10) code for nontraumatic spinal hematoma.

Patients’ records, imaging data, laboratory results and surgical reports were analyzed.

Medical history was extracted from both medical records and questionnaires completed by patients and their families. The neurological status was documented daily. The modified Rankin Scale (mRS) was utilized to assess outcomes at discharge and during follow-up.

### Statistical analysis

Statistical analysis was perfomed by SPSS Version 29.0.1.0. For the descriptive analysis, data is presented as Mean and standard deviation (STD) or Median and range. The Kolmogorov–Smirnov test was employed to assess the normal distribution of variables. The predetermined level of statistical significance for all tests was established at p < 0.05.

### Ethical approval

The study was approved by the Research Ethics Committee of the Technical University of Munich (2023-630-S). Informed consent was not required for all patients due to the retrospective design in accordance with local ethics committee protocols. However, informed consent was obtained for all case reports. The study adhered to the tenets of the Declaration of Helsinki. No funding was received.

## Results

### Patients

**Fig. 1:**
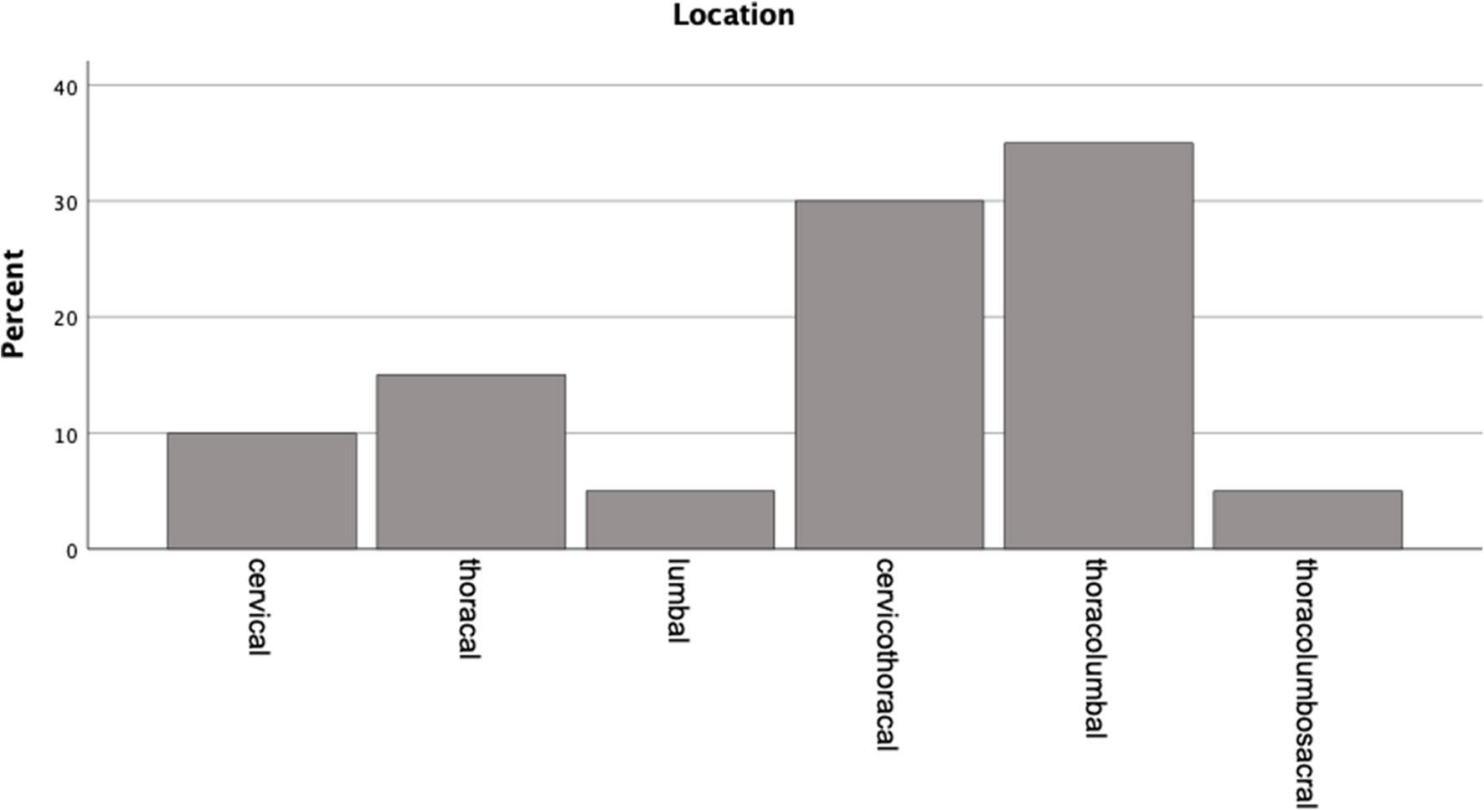
Distribution of location of hematomas. The spinal level distribution of affected individuals deviates from normality (p = 0.002, Kolmogorov–Smirnov test)

**Fig. 2:**
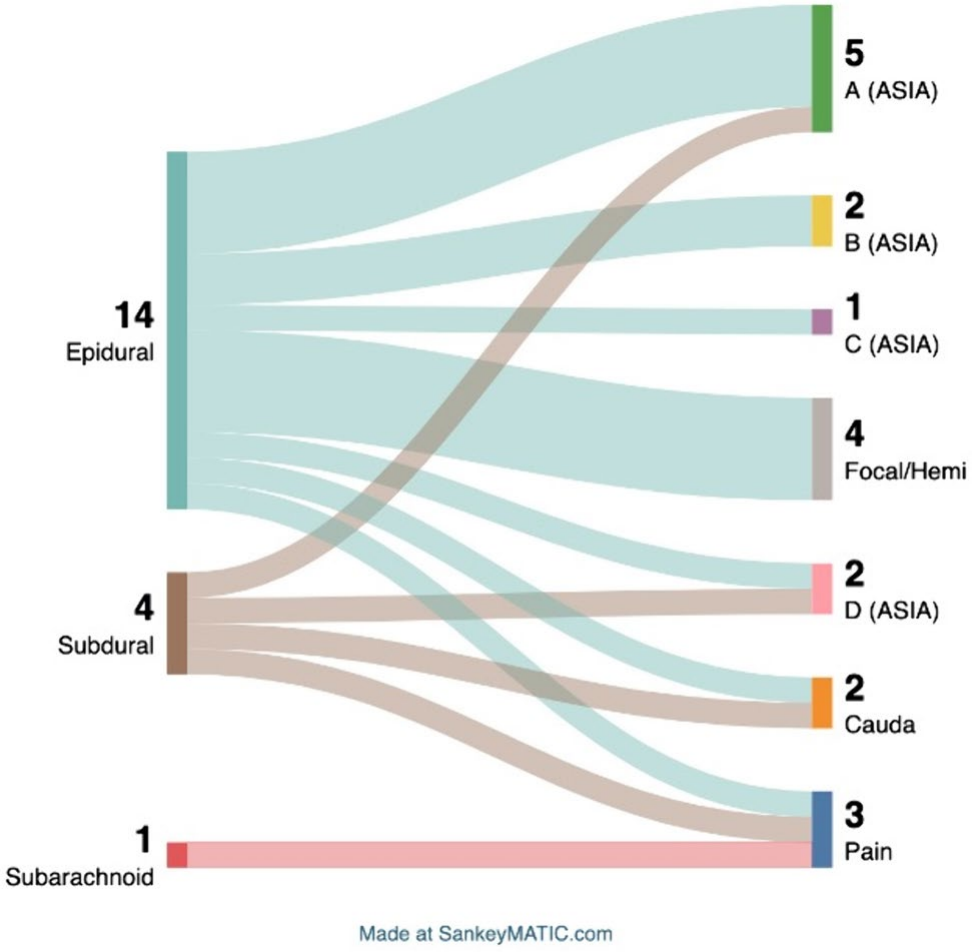
Location of hemorrhage and associated symptoms. Note: one bleeding was combined subdural and epidural and is not included due to graphical design

We enrolled 20 patients of a median age of 66 (range 39—85) years treated between 01/01/2017 and 11/15/2023 at our department for spontaneous spinal hematoma (Table 1). Nine (45%) were male, eleven (55%) were female. Hematomas were distributed as follows: three in the cervical region, six in the cervicothoracic region, two in the thoracic region, seven in the thoracolumbar region, one in the lumbar region, and one in the thoracolumbosacral spine (Fig. 1). Among these cases, 14 were epidural hematomas, four were subdural hematomas, one was a combined epidural and subdural hematoma, and one was a subarachnoid hematoma (Table 1, Fig. 2). A total of 17 patients (85%) presented with a neurological deficit, while three patients experienced symptoms solely related to pain. Five patients had ASIA A spinal cord injury, two had ASIA B, two had ASIA C and two had ASIA D. Three patients presented with armparesis, one with hemiparesis and two with cauda equina syndrome (Table 1, Fig. 2).

**Table 1:** Neurological deficits in relation to location of the bleeding and etiology. Absolute numbers are displayed

Neurological impairment	Location (n)	Etiology (n)
ASIA Impairment scale (11)		
A (5)	epidural (4) subdural (1)	coagulation therapy (4) no identified cause (1)
B (2)	epidural (2)	coagulation therapy (2)
C (2)	epidural (1) combined* (1)	vascular malformation (1) coagulation therapy (1)
D (2)	epidural (1) subdural (1)	coagulation therapy (1) no identified cause (1)
Other deficits (6)		
Focal or hemiparesis (4)	epidural (4)	coagulation therapy (2) no identified cause (2)
Cauda equina syndrome (2)	epidural (1) subdural (1)	coagulation therapy (1) vascular malformation (1)
Pain (3)	epidural (1) subdural (1) subarachnoid (1)	vascular malformation (3)

*combined epidural and subdural

### Reasons for spinal hematoma, diagnostics, and treatment

**Fig. 3:**
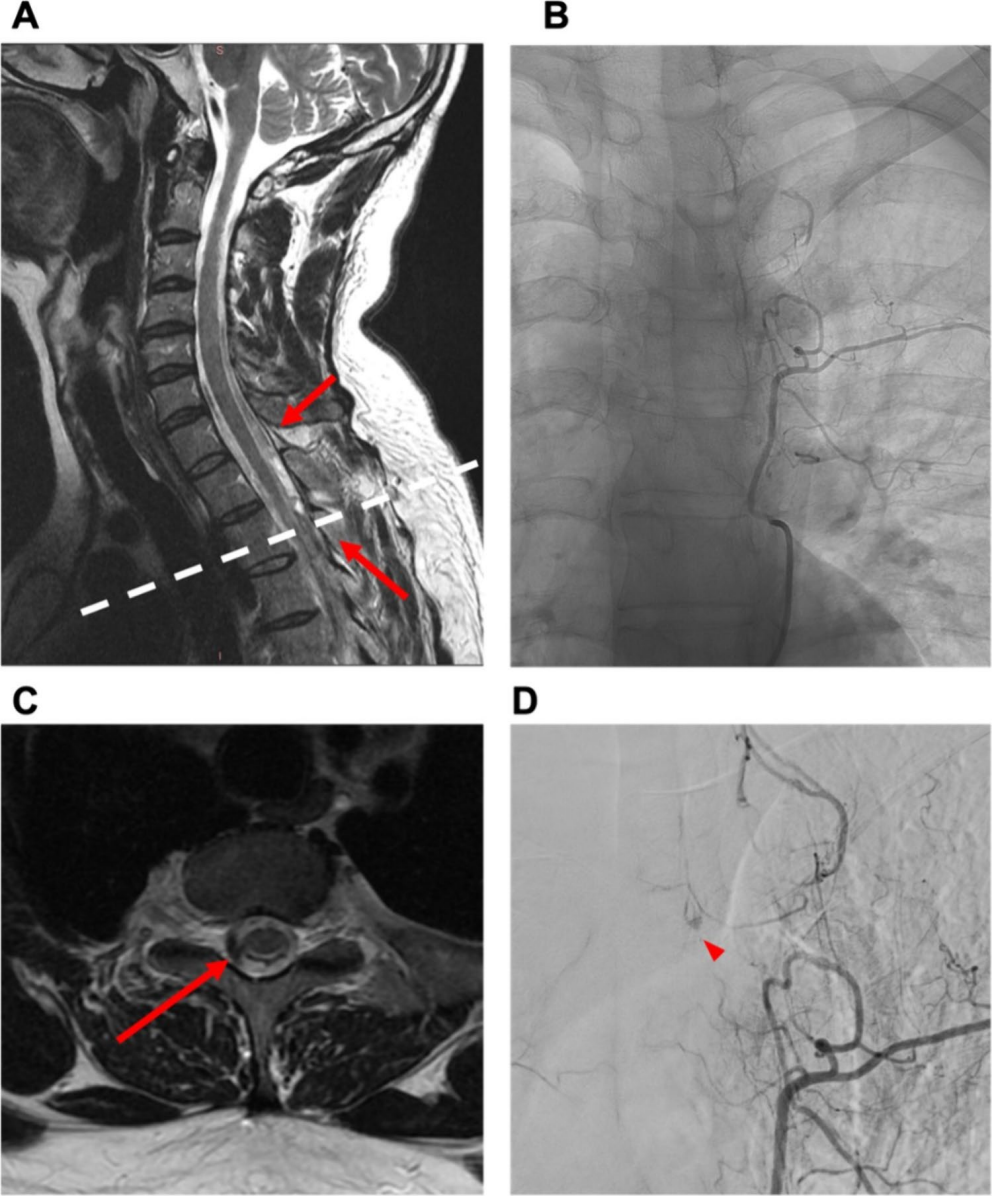
Case 1: 39-year-old man with spontaneous spinal subdural hematoma. A 39-year-old male patient presented to the emergency department ten days after a COVID infection, reporting symptoms such as fever, fatigue, and back pain persisting for one week. The patient had experienced intermittent episodes of back pain, self-treated with aspirin as needed before seeking medical attention. Subsequently, the patient developed a severe headache and meningism. There was no history of trauma, coagulation disorder, or other pre-existing diseases. Coagulation and infection parameters in the laboratory results were observed to be within typical ranges. Neurological examination revealed, except for meningism, no further neurological deficits. Gadolinium-enhanced MRI and MR angiography (MRA) identified the presence of subdural hematoma along the cervical and thoracic spine, initially without a clearly identified structural source of bleeding (**A**). Additional spinal DSA was conducted, revealing a small suspicious lesion situated within the left lateral spinal canal at the T3/4 level (**B** + **D**). Surgical intervention, specifically a hemilaminectomy at T3 and T4 on the left side, was conducted. No definite vascular malformation was found except for pathologically coagulated veins. Postoperatively, the patient presented with hemihypesthesia below T6 on the left side, proximal paresis of the left leg (MRC 3/5), and bladder/bowel dysfunction. A subsequent gadolinium-enhanced MRI revealed residual pathological hypervascularization at T3/4 on the left (**C**). A re-operation with an extended approach was performed as follows: The midline skin incision was reopened, and the left L3 hemilaminectomy was extended cranially. The dura was reopened, and a likely thrombosed hemangioblastoma with associated vessels was exposed and excised. Motor-evoked potentials remained stable throughout the procedure. Following the second surgery, the patient manifested without further neurological deficits as previously described. Postoperative digital subtraction angiography revealed no residual vascular irregularities and histopathologic examination identified the lesion as vascular malformation. He was transferred to a rehabilitation center on postoperative day (POD) 2

**Fig. 4:**
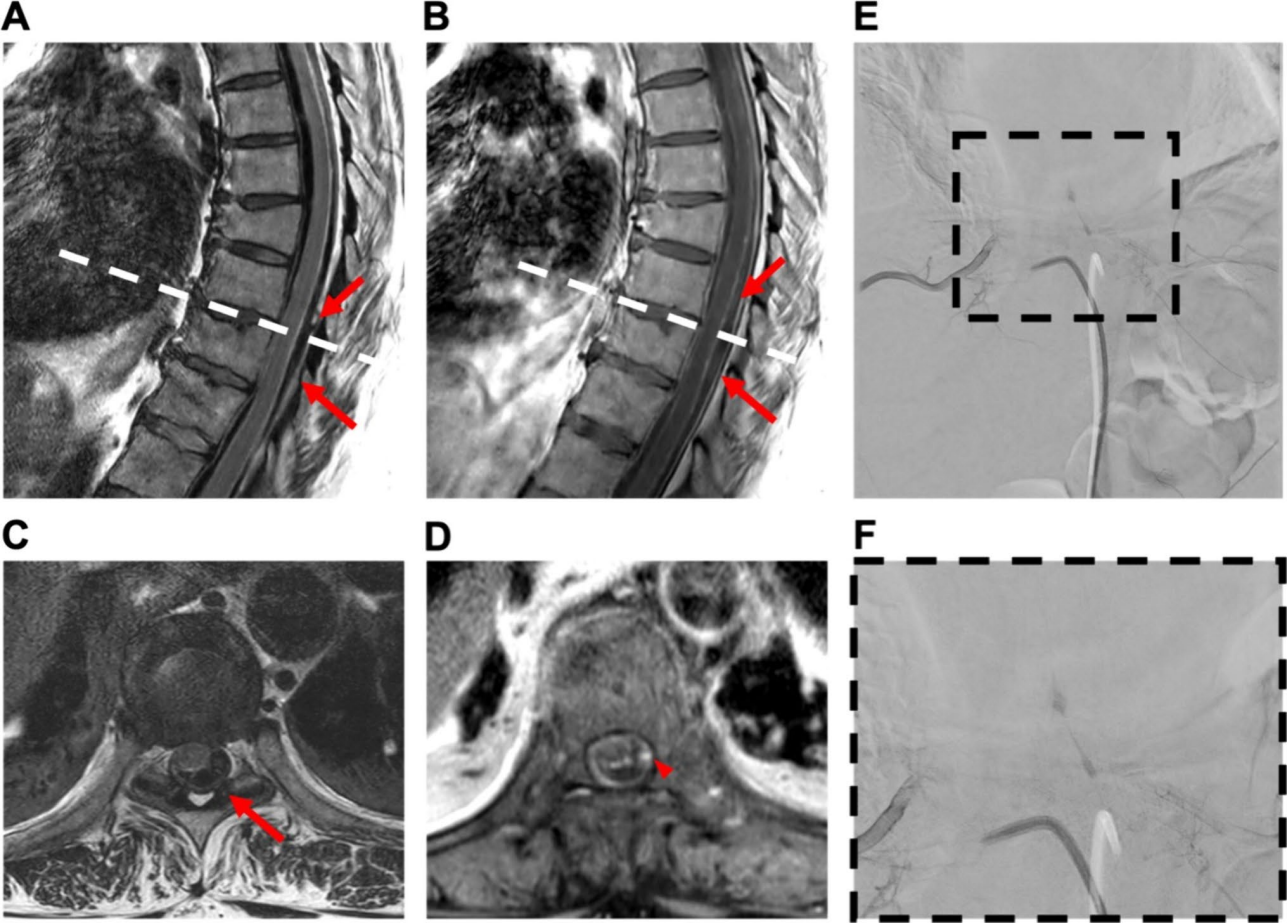
Case 2: 61-year-old woman with spontaneous spinal epi- and subdural hematoma. A 61-year-old female patient was referred from a community hospital with symptoms of generalized pain, primarily localized in the upper abdomen, and paraparesis (MRC 3/5) without sensory deficits. Laboratory findings indicated the absence of anomalies in coagulation parameters, with a slight increase observed in infection markers. Furthermore, cardiac parameters were elevated, including cardiac troponin, creatine kinase, and its cardiac isoform (CK-MB). Despite the elevation, myocardial infarction was excluded, and the increased levels were attributed to mild cardiac decompensation. Gadolinium-enhanced spinal MRI disclosed a hemorrhage classified as epidural extending from T6 to L2 without a clearly identified structural source (**A** + **B**). Emergency evacuation of the epidural hematoma was performed through left-sided interlaminar fenestrations at T4, 6, 8, and 10, along with hemilaminectomy at L2. Postoperatively, there was no improvement in neurological function, but pain intensity decreased. Subsequent MRA revealed discrete contrast enhancement at the T9/10 level on the left lateral intraspinal region (**C** + **D**). Additional DSA confirmed a suspicious structure on the left side at the T9/10 level (**E** + **F**). The patient underwent a second surgery: The midline skin incision was reopened. Hemilaminectomy of T9 and T10 on the left-hand side was performed with undercutting to the opposite side. A recurrent hematoma was completely removed from the epidural space. The dura was opened and an additional intradural hematoma was evacuated. A vascular anomaly arising from the segmental artery was identified, resulting in elongation of the perimedullary vessel. The vessel was carefully coagulated, transected, and sent for histopathological examination. Motor-evoked potentials were not obtainable from the beginning of the surgery. Following the second surgery, paralysis of the left leg was observed, while the paresis of the right leg resolved. Gadolinium-enhanced MRA and DSA conducted after surgery verified the complete elimination of the AVM. Histopathologic examination confirmed the presence of an AVM. At discharge to a rehabilitation facility, there was a modest improvement in the motor impairment of the left leg, characterized by subtle movements of the foot but no motor function in the proximal leg

**Fig. 5:**
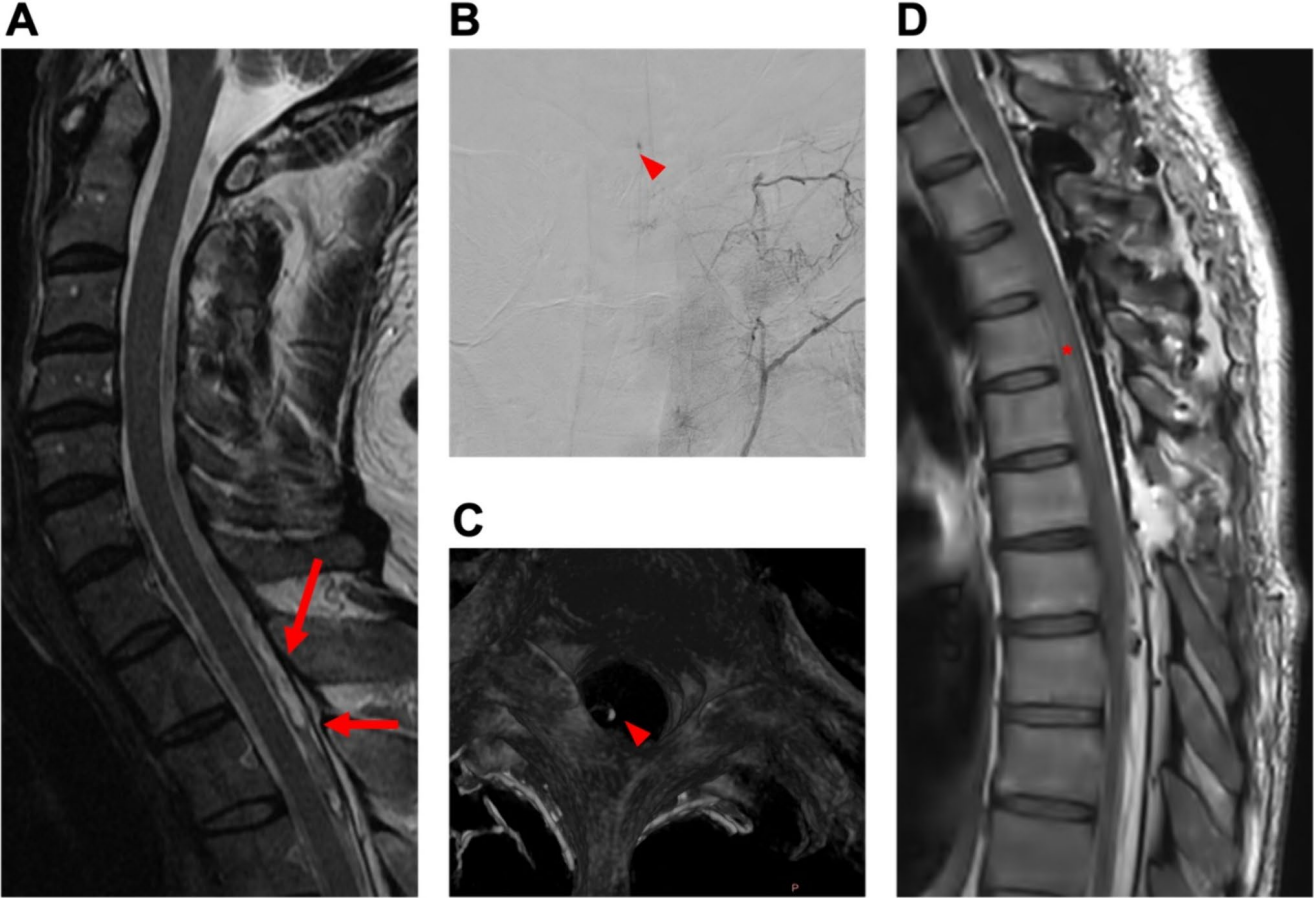
Case 3: 59-year-old woman with spontaneous spinal subarachnoid hemorrhage. A 59-year-old female patient with sudden-onset severe headache and intense neck pain, indicative of suspected subarachnoid hemorrhage (SAH), was referred to our department. Neurological examination showed, except for meningism, no further neurological deficits. Cranial Computed Tomography (CT) with angiography and cranial gadolinium-enhanced MRI were conducted to exclude SAH. Subsequent spinal MRA revealed spinal subarachnoid hemorrhage along T3-8 with contiguous myelomalacia and minor hemorrhages distributed at levels S3-5, initially without a clearly identified source of bleeding (**A**). The patient was not under anticoagulant medication and had no history of coagulation disorder or trauma. Laboratory findings revealed no abnormalities nor indications of infection. Spinal DSA revealed the presence of a small, arterialized vessel entering the intraspinal region on the right side at the level of T8 (**B**). First, laminoplasty on T3-6 (where the punctum maximum of the hematoma was) and evacuation of the hemorrhage were performed. However, no obvious vascular malformation was identified. Immediately postoperatively, the patient exhibited only mild right-sided proximal leg weakness (MRC 4/5). However, overnight, this deteriorated to paralysis. Postoperative MRA ruled out re-bleeding, but additional DSA revealed a suspicious vascular lesion at the T3 level. Another spinal MRA disclosed the presence of a nodular lesion, consistent with DSA findings, within the dorsal median extramedullary space at the T3 level (**C**). Subsequently, the patient underwent reoperation, extending the approach: The previous skin incision was reopened. Laminoplasty was extended cranially by one level (T2). The dura was opened, and small pathological vessels along the right T2 and T3 nerve roots were identified, resected, and submitted for histological examination. Motor-evoked potentials remained stable on the left side but were absent on the right. The histopathological assessment of intraoperatively obtained samples corroborated the diagnosis of an AVM. Following the second surgery, although the right leg paralysis persisted, there was noticeable motor deterioration in the left leg (MRC 2/5). Additional symptoms encompassed sensory deficits below T5 and bladder dysfunction. Gadolinium-enhanced MRI effectively ruled out rebleeding but revealed an extended thoracic myelomalacia (**D**). Subsequent DSA did not find any other vascular abnormalities. The motor function of the left leg gradually improved, reaching a proximal MRC of 4/5 and a distal MRC of 5/5 over time. Hypesthesia below the T5 level and bladder dysfunction endured, along with persistent paralysis in the right leg. The patient was referred to a rehabilitation facility

**Fig. 6:**
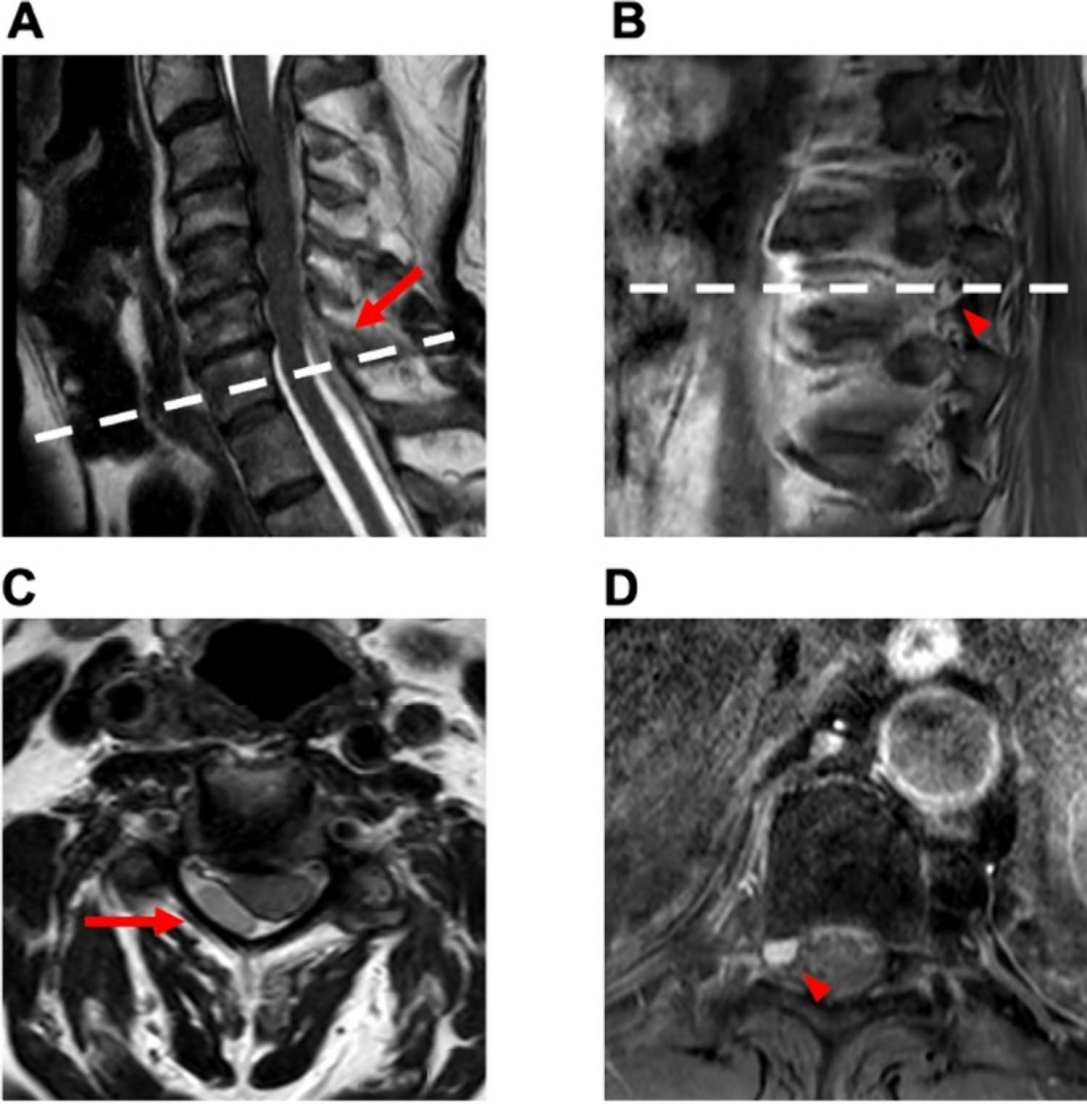
Case 4: 66-year-old man with spontaneous epidural hematoma. A 66-year-old male presented to the outpatient clinic for clinical and imaging follow-up. One month earlier, he had consulted the emergency department, experiencing pain between the shoulder blades. MRI revealed a spontaneous epidural hematoma from C2 to T3 (**A** + **C**), which was treated conservatively. At follow-up, the patient reported a complete resolution of his pain. A subsequent MRI showed a contrast-enhancing lesion at the T9 level on the right side (**B** + **D**). Upon discussion of the case in our neuro-oncology tumor conference, a resection of the lesion was recommended due to suspicion of a tumor. A preoperative DSA was not performed. The patient had no neurological deficits, and laboratory results were normal. Elective microsurgical excision of the extradural lesion was performed without any postoperative complications. The patient exhibited no sensorimotor symptoms. Histopathological examination confirmed the lesion as a hemangioma. The patient was discharged on the second postoperative day

Of the total cohort, eleven (55%) patients were under anticoagulant medication (Table 2). One patient was under anticoagulant medication and had undergone a lumbar puncture before MR imaging for diagnosis was conducted. However, the patient was already admitted to the hospital with symptoms attributed to the spinal bleeding. Conclusively, the lumbar puncture was part of the diagnostic assessments and was not seen as cause of the hemorrhage. We present four representative cases with spinal hematoma (Figs. 3, 4, 5, and 6)

**Table 2:** Etiology of spinal hematomas

Etiology	N (%)
Anticoagulant medication	11 (55)
*Type of anticoagulant*	
Coumarin	3 (27)
NOAC	3 (27)
Aspirin	2 (18)
Clopidogrel	1 (9)
Coumarin + Aspirin	1 (9)
NOAC + Aspirin	1 (9)
Vascular anomality	5 (25)
*Type of vascular anomality*	
AVM	3 (27)
Cavernoma	1 (9)
Hemangioblastoma	1 (9)
Idiopathic	4 (20)

In four (20%) cases the cause of the spinal bleeding remained unclear. Vascular anomalies were identified in five (25%) cases including three cases of arteriovenous malformation (AVM), one case of hemangioblastoma, and one case of cavernoma. Digital subtraction angiography (DSA) was performed in four (80%) of these cases. However, as emergency surgery was required in two cases due to spinal cord compression symptoms, DSA could only be conducted preoperatively in these instances. In the two cases that underwent preoperative DSA, it revealed suspicious vascular structures, albeit without clear certainty. Magnetic resonance imaging (MRI) was performed for all patients. 18 patients were treated surgically, while two patients were treated conservatively, due to the absence of neurological deficits and spontaneous regression of the epidural hematoma. Notably, one conservatively treated patient with a cervical epidural hematoma revealed a structure on Th9 in the MRI, initially presumed to be a neurinoma. Resection of this structure was performed at intervals. Intraoperative and histological findings revealed a cavernoma (Fig. 6).

### Outcome

Mean preoperative MRC level was 2.3 (STD 1.8). In total, there was only a marginal improvement to a mean MRC level of 3 (STD 1.9) (p = 0.164) postoperatively. However, in relation to individual cases, postoperatively, 13 out of 20 (65%) showed improvement in their neurological function, four (20%) maintained their level, and only three (15%) experienced deterioration. Follow-up rate was nine out of 20 (45%) with a mean follow up period of 2.1 months (STD 2.0). At the last follow-up 45% showed an improvement of their neurological function.

## Discussion

### Demographics and localization

The incidence of spinal hematoma is low. Kreppel et al*.* [12] described only 613 published cases over 170 years. At our department, which is a Level I spine center, we identified 20 cases over a period of almost six years. Generally, spinal epidural hematomas are the most prevalent, followed by subarachnoid hematomas [12]. However, within our department, the predominant instances were epidural hematomas, with subdural hematomas ranking as the second most common. The median age at presentation was 66 years, consistent with findings reported in previous studies [12] which indicated two peaks: one between 15–20 years and another between 45–75 years. A male-to-female ratio of 2:1 has been reported for all intraspinal hematomas [12], while subdural hematomas show a slight female predominance [4]. Our results indicate a slight predominance of female patients with respect to all spinal hemorrhages. However, it is important to note the small sample size of our study. Statements regarding the most frequent localization of spinal hematomas vary among sources, but generally include the cervicothoracic, thoracic, and thoracolumbar regions [4, 6, 12, 20], as observed in our study. Subdural hematomas are predominantly located ventrally to the spinal cord or encircle it with a significant ventral component, whereas epidural bleedings are located dorsally to the dural sac [13].

### Etiology

In the majority of cases spinal hematomas originated in patients under anticoagulant therapy. Spinal hematomas with a structural vascular cause, as well as those without an identifiable cause, were rare. In literature, idiopathic cases are described to account for up to 30 – 40% of cases [6, 12]. The pathomechanism of spontaneous idiopathic spinal hematomas is still a matter of debate. While some authors postulate that the posterior epidural venous plexus is the most probable source [7], others attribute the hematoma's origin to an arterial rupture [2]. Furthermore, depending on the subtype of hematomas, theories of origin vary. For instance, some authors speculate that a spinal subdural hematoma is a result from an extension of a subarachnoid spinal hemorrhage, given that the subdural space is a potential avascular compartment [11]. Others suggest that unrecognized vascular anomalies are responsible for intraspinal hematomas [17–19]. The inability to detect these malformations preoperatively might be associated with either the small size of these anomalies or thrombosis following the initial bleeding episode [7]. Previous studies describe cases of spontaneous spinal hematomas with initially negative DSA findings but abnormal vascular structures found intraoperatively: For example, Akutsu et al*.* [1] reported a case of spontaneous epidural hematoma in a 27-year-old man with abnormal epidural veins resembling varices found intraoperatively and confirmed histopathologically. Dawson et al*.* [3] documented two cases of spontaneous epidural hematoma attributed to "venous angiomatous malformations”. Yu et al*.* [26] found “dilated and engorged vascular structures” intraoperatively which was confirmed by histopathologic analysis despite negative preoperative DSA in nine patients.

The three exemplary cases described above also highlight that despite an initially negative MRI finding and only questionable results in the DSA, a vascular pathology underlay the spinal hemorrhages. Additionally, the second case underscores the challenge of detecting subdural hematoma on MRI, as it is isointense to cerebrospinal fluid and the spinal cord [13], or may be masked by a concomitant epidural hematoma, as observed in this instance.

However, it is important to distinguish the etiology of spinal hematomas. As mentioned above, spinal vascular malformations are very rare. In most cases of spinal hematomas, regardless of their exact location, anticoagulant therapy is the primary cause. In 30–40% of cases, the cause remains unknown.

### Symptoms and treatment

Acute and rapidly progressive neurological deficits can arise from a diverse range of etiologies. The presence of painful paraparesis should direct attention toward a potential spinal pathology [13]. The possibility of epidural, subdural, and subarachnoid spinal hemorrhage, even in patients without history of recent trauma and normal blood coagulation parameters, should always be taken into consideration. Particularly noteworthy is the potential for spinal hematoma to manifest in diverse ways, resembling intracranial subarachnoid hemorrhage with features such as meningism, altered consciousness, or epileptic seizures as it was shown in the first and third case. However, the majority of our patients presented with pain and neurological deficit. Most patients were treated surgically, as it is the state of the art for patients showing signs of progressive spinal cord compression [10]. Decisive factors for recovery after spontaneous spinal hematoma with symptoms of spinal cord compression are the preoperative neurological deficit's severity and the time elapsed until decompression is performed [8].

Despite the emphasis on surgical treatment, there are reports of successful conservative approaches: Lefranc et al*.* [14] described a case of traumatic cervical epidural hematoma, initially presenting with a 2/5 paresis of the deltoid, biceps and triceps, which spontaneously improved within a few hours and completely resolved after six days without surgical decompression. Wagner et al*.* [23] reported a case of cervical epidural hematoma in a patient under continuous aspirin medication. The patient initially presented with proximal arm paralysis, which showed improvement within 12 h and complete recovery after three days. These authors advocate a conservative treatment when patients show a stable or improving neurological status. In our study, only two patients were treated conservatively as they lacked neurological deficits, and the epidural hematoma regressed spontaneously as confirmed by follow-up MRI. For both cases an etiologic factor for the hematoma was identified. However, in cases like the ones presented above, where there is no neurological deficit, but the etiology of the spinal hematoma remains unclear, comprehensive diagnostics should be undertaken, ultimately leading to surgical exploration. This is necessary to potentially identify and remove vascular pathology as the cause of bleeding, thereby preventing the progression or recurrence of the hematoma. However, elaboration of the right surgical strategy is an important factor for success. Particularly in emergency settings, the choice of surgical strategy is influenced significantly by the surgeon's experience. Surgeons with more experience may opt for more complex procedures or approaches that require more technical skill. Conversely, less experienced surgeons might choose more straightforward techniques to ensure patient safety and achieve the best possible outcome. In the emergency setting, a less experienced surgeon might concentrate on evacuating the hemorrhage while diagnosing and eliminating the underlying vascular malformation might be reserved for a later time point in a second stage (see case 2). Additionally, the patient's symptom load, including the progression of neurological deficits, also plays a critical role. Urgent surgical intervention may be required in cases of rapid neurological deterioration or impending neurological compromise. For example, in Cases 2 and 3 of our study, the origin of bleeding was not definitively identified during the initial diagnostic assessment. In these patients, the hematomas were evacuated at the site where they were most extensive. Subsequently, advanced imaging modalities were employed to detect the vascular lesion, leading to planned secondary surgical intervention.

## Conclusions

Spontaneous spinal hematomas are a rare but severe neurological disorder, demanding immediate diagnostic assessment and intervention to prevent lasting neurological impairment.

The findings highlight the importance of comprehensive diagnostics and, finally, surgical exploration, especially in cases with unclear etiology, to identify and address vascular pathology and prevent hematoma progression or recurrence. The choice of procedure and approach must be carefully weighed against the situation's urgency and the potential risks associated with surgery. In summary, both the surgeon's experience and the patient's clinical presentation are critical factors influencing the selection of the optimal operating strategy in SSH.

## Data Availability

Any authorized researcher making a reasonable request may contact the corresponding author to obtain anonymized data.

## Abbreviations

DSA: – Digital subtraction angiography
ICD-10: – International Classification of Diseases, Tenth Revision
mRS: – Modified Rankin Scale
AVM: – Arteriovenous malformation
MRI: – Magnetic Resonance Imaging
NOAC: – Novel oral anticoagulant drug
MRC: – Medical Research Council’s scale of Muscle power
POD: – Postoperative day
ASIA: – American Spinal Injury Association Impairment Scale
STD: – Standard deviation
MRA: – Magnetic Resonance Angiography
CT: – Computed tomography
SAH: – Subarachnoid hemorrhage

## Code availability

We did not use any custom computer code or algorithm to generate results. Statistical analysis was performed using SPSS Version 29.0.1.0.
